# Making the BEST Decision—the BESTa Project: Description of the Design and Alpha Phases as Part of the Development of a Digital Decision Aid for Cancer Screening in Sweden

**DOI:** 10.1007/s13187-025-02633-y

**Published:** 2025-04-26

**Authors:** Kaisa Fritzell, Johanna Wangmar, Berith Hedberg, Anke Woudstra, Anna Forsberg, Anders Kottorp, Karl A. Franklin, Anna Jervaeus

**Affiliations:** 1 Department of Neurobiology, Care Sciences and Society, Division of Nursing, Karolinska Institutet, Alfred Nobels Allé 23, 141 52 Huddinge, Sweden; 2 Theme Cancer, Hereditary Cancer Clinic, Karolinska University Hospital, Stockholm, Sweden; 3 School of Health and Welfare, Jönköping University, Jönköping, Sweden; 4 Team Advies en Onderzoek, Municipal Health Service (GGD) Kennemerland, Haarlem, the Netherlands; 5 Department of Medicine Solna, Division of Clinical Epidemiology, Karolinska Institutet, Stockholm, Sweden; 6 Faculty of Health and Society, Malmö University, Malmö, Sweden; 7 Department of Diagnostics and Intervention, Umeå University, Umeå, Sweden

**Keywords:** Early detection of cancer, Health promotion, Public health, Decision Making, Shared, Decision support techniques

## Abstract

A digital decision aid for cancer screening can gather balanced information in one place and give individuals the opportunity to elucidate their knowledge, values and engage in shared decision-making. Research shows that ethnic minorities and individuals with various functional limitations participate in cancer screening to a lower extent, hence our ambition to make the decision aid as available and applicable as possible, regardless of end-users’ traits. The aim was to describe the *design* and *alpha phases* of the development of a digital decision aid for CRC screening and tentative end-users’ perceptions. Based on a scientific framework for development of decision aids, participants were recruited through multiple channels. The decision aid was evaluated in two steps, a paper version (design phase) and a website prototype (alpha phase), using the think-aloud approach. Data were rich with detailed suggestions for improvements of the decision aid and how it was perceived. A positive outlook on the decision aid was common. Certain words, wordings or visual features were considered difficult and worry or anxiety, related to the content, were expressed. The variation in the findings illustrates the challenges of decision aid development. Still, our findings emphasise the importance of designing a decision aid in co-creation with its end-users. Developing a digital decision aid is complex, why a well-established framework is essential. With the goal of an equal healthcare system, the inclusion of individuals with diverse backgrounds and functional limitations should not only be a fundamental aspect of all research, but a prerequisite.

## Introduction

As a large amount of the global annual cancer incidence occurs in Europe, the European Commission has initiated the development of the European Code Against Cancer [1], first published in 1987 and updated in the 4 th edition in 2014 [2]. It includes actions to reduce cancer risk on an individual level such as participation in organised screening to prevent colorectal cancer (CRC) and breast and cervical cancer [2]. A digital decision aid (DA) for cancer screening [3] facilitates the presentation of balanced information in one place and gives individuals the opportunity to elucidate their knowledge, values and preferences as well as to discuss their decision with healthcare professionals, i.e. engage in a shared decision-making (SDM) process [3]. This will hopefully lead to individuals making autonomous and informed decisions regarding screening participation, in line with their values and preferences, including those known to participate in cancer screening to a lesser extent. Research shows disparities in participation related to socioeconomic status [4]. An equal and health-promoting healthcare system is crucial but is not the reality today. Our ambition with the digital DA is to move towards a more equal system and an improved ethical practice by making the DA as available and applicable as possible, regardless of end-users’ background, physical abilities or mental health status.

## Background

Participation in a screening programme increases the possibility of reducing mortality and morbidity for that particular disease in the population [2], by finding cancer at an early and treatable stage, as well as removing precancerous lesions. Recent data from a large Swedish cohort study (from 2008 to 2021) found a 14% decrease in CRC mortality, connected to screening (faecal test) [5]. In Sweden, The National Board of Health and Welfare states that screening should be offered (for cervical and breast) [6] and recommended (for CRC) [7] to all eligible individuals, free of charge. The DA is intended to serve as a pedagogical platform, for both knowledge and educational purposes, directed to all interested parties, especially those invited to screening. Providing relevant information on the benefits as well as risks of screening should not be in conflict with screening participation [8]. The DA should inform, educate and raise awareness rather than persuade. DAs usually provide evidence-based information about options, associated benefits, harm and probabilities and aim to guide individuals to recognise their personal values associated with the decision [3, 9]. Health literacy, a multi-dimensional concept including numerical skills, information seeking, decision-making and critical thinking [10], is considered central as an asset to improve individuals’ empowerment regarding disease prevention and health promotion [10]. In Sweden, screening decisions are expected to be made by the individual himself or herself at the time the invitation arrives in the letterbox. The tradition of seeking preventive healthcare advice is rare. A DA may therefore help to facilitate a SDM [11] process if so desired, between the individual and a healthcare professional [3]. Nevertheless, at present, no DAs exist in Sweden related to screening decisions.

Related research from our group on CRC screening revealed few differences, between those who chose to participate in screening and those who declined, regarding health literacy [12] and anxiety, in relation to the decision [13]. Both participants and non-participants reported limited knowledge but differed regarding values and preferences [14, 15]. In addition, a recent review found lack of knowledge regarding the faecal test, technical problems with the test kit, little knowledge about screening and low health literacy as barriers towards adherence to CRC screening [16].

Regardless of the statement made by The National Board of Health and Welfare in Sweden, it is known that socioeconomic status plays an important role in CRC screening participation [4]. Furthermore, individuals from ethnic minorities and those with various limitations (e.g. functional limitations, mental health problems) participate in cancer screening, or specifically CRC screening, to a lesser extent [16–18]. In line with this, a recent evaluation found racial and ethnic inequities regarding participation in oncology clinical trials [19]. Moreover, a review from 2023 [20] concluded that lower screening rates (faecal occult blood test) among culturally and linguistically diverse (CALD) populations might be due to the fact that screening programmes do not meet their individual needs. Nevertheless, educative group sessions and narrative-based screening information increased participation [20]. In order to work towards an equal and health-promoting healthcare, it is essential to work inclusively with an end-user approach, bearing in mind the large global differences in CRC rates (highest in Europe and lowest in Southern Asia) and the fact that CRC is highly preventable [21].

This project is based on the International Patient Decision Aid Standards (IPDAS) framework [22, 23] and the suggested model for the DA development process by Coulter et al. [24]. In the process, lay people are included from the very beginning, and such approaches have previously been taken in similar projects from other countries [25, 26]. Briefly, the framework consists of five steps (described in more detail in previous work (27) and in the ClinicalTrials.gov identifier NCT05512260, registered August 2022): (1) *define scope* (a digital DA directed towards individuals invited to any of the three screening programmes (CRC, breast, cervix) in Sweden); (2) *form a steering group* (consisting of lay people, researchers and clinicians with different backgrounds and professions); (3) *conduct the design phase* (with the DA in paper format to be evaluated by experts and lay people); (4) *alpha testing* (with the prototype of the digital DA to be evaluated by lay people and experts); (5) *beta testing* (with the DA to be evaluated in real-world setting).

With this background, the overall aim of the present project is to develop, implement and evaluate a digital individual decision aid for people invited to cancer screening in Sweden, starting with CRC and later expanded to other cancer diagnoses included in screening programmes. The specific aim of this study is to describe the *design* and *alpha phases* of the development of an individual digital decision aid for CRC screening and the perceptions of tentative end-users.

## Methods

### Overall Design

This project encompasses a process, implementation and outcome evaluation of the DA, presented in more detail in the protocol [27]. Since this work represent an iterative process, the *design phase* (A) will be described first, including both methods and results, and thereafter the *alpha phase* (B).

### The Design Phase

#### The Decision Aid (DA)

A DA has been developed, based on literature reviews and inspired by the work of Schwartz and colleagues [25] and discussed in the steering group for the project. The DA, in paper format, includes the following: (2) a section on how to make decisions and how a DA can provide guidance; (2) information on screening, cancer screening, screening tests and the specific disease (initially CRC but later on to include more cancer diagnoses eligible for screening), benefits and harm; (3) values and preferences; (4) a life-style chapter on physical activity, diet and alcohol; (5) questionnaires with items on values and preferences, participation, cancer screening knowledge, involvement, lifestyle, evaluation of the DA and background data. The DA also contains both abstract and realistic pictures to illustrate the text or main message. Ultimately, the DA will be made public and accessible online from a laptop, smartphone or tablet to all who are interested in cancer and cancer screening in Sweden.

#### Participants and Study Setting

Participants were recruited via purposive sampling through multiple channels and by, to some extent, applying the snowball method [28]. Individuals with any self-reported functional limitations were approached via information distributed by different patient organisations (non-governmental organisations (NGOs)). Certain experts were also approached via patient organisations or RCC (regional cancer centres). For instance, health informants (working with education and information towards immigrants in Sweden) with different ethnic backgrounds were recruited via RCC. Inclusion criteria: individuals eligible for CRC screening, i.e. women and men aged 60–74 from the whole of Sweden and experts on specific areas of interest. No further stratification was done, such as being at risk for CRC, or other aspects. One pilot interview was performed by KF before the actual data collection started to test the think-aloud approach [29] and the pre-defined documentation sheet. After the interview, it became clear that the chapter on values, preferences and attitudes needs to be expanded. In accordance with feedback from the participant, the language was also revised in the DA (e.g. adjustments to sentences that were too long).

#### Data Collection

Relevant organisations (NGOs, RCC) and individuals were approached through e-mail, and if they failed to respond, they were then contacted by phone. Information about the research was distributed to those individuals or NGOs expressing an interest. After that, and for those still interested, a timepoint for an individual interview was booked in accordance with participant’s preferences. Prior to the interview, the DA, in paper format (ca 30 A4 pages including pictures), was distributed to those who wanted to read it. The interviews were performed via Zoom®/Microsoft Teams® with one author (AJ) performing the interview and another author (KF) taking notes according to a pre-organised Excel® sheet (based on the topics in the DA, please see “The Decision Aid” section) inspired by earlier research [30]. The interviews were not audio-recorded as we used such a detailed protocol for documentation. Each participant took part in one interview, and a sign language interpreter was offered when needed. The interview followed the structure of the DA with the help of an interview guide with some broad questions, and the participant was asked to think-out-loud [29] and comment on aspects such as content, wording, missing information and pictures. Follow-up questions were sometimes asked for clarification, such as can you present an alternative wording, or can you give an example on how this could be presented instead. The questions regarding knowledge, values and preferences, involvement, lifestyle, participation and evaluation of the DA were scrutinised by the participant, also using the think-aloud approach. Questions on knowledge, values and preferences are derived from the National Survey of Medical Decisions (the DECISIONS study) [31] which we translated and culturally adapted into a Swedish version [32] and evaluated psychometrically [33]. However, the psychometric evaluation revealed that the translated and adapted questionnaire lacked sound psychometric properties [33]. Consequently, the decision was made to include selected relevant items (with some modifications) from the questionnaire, for further evaluation in the TA sessions. The self-reported data collected included sex, accommodation, highest level of education, occupational status and previous screening experience. All participants were given two cinema tickets as a small incentive. The recruitment period started during spring 2022 and ended during autumn 2023.

#### Data Analysis

The data from all interviews were analysed by using a combination of *summative content analysis* and *conventional content analysis*, both described by Hsieh and Shannon [34].

- The *summative approach* included sorting and quantifying words or statements to explore the usage. All statements were colour-coded according to similarities and then grouped and labelled by content. We have previously used a similar approach when analysing phone calls to a help line regarding screening participation [30].
- The *conventional approach* included inductive coding derived from the data, after which the codes were sorted and organised into categories (Table 2).
- The *pictures and items* in the DA were analysed by colour-coding all individual statements, about each picture/item. Different colours were used to indicate various types of content. Thereafter, the content of each colour group was organised and labelled according to content (Tables 3 and 4).

### Findings from the Design Phase

#### Background Characteristics

The number of interviews was 18, of which 14 were online and 4 in person. The interviews occurred between February to October 2023 and varied in length from 57 to 155 min. Among those participating, 14 had read the DA beforehand, whereas 4 had not, and 16 provided feedback on the pictures. One participant had a sign language interpreter during the interview and two used their visual aids. The pilot interview is included in the final sample. Background characteristics are presented in Table 1. Country of origin varied among participants, and countries in Asia, Europe and South America were represented.

**Table 1:** Background characteristics of participants in the design phase

	Participants *n* = 18
Gender	
Women	11
Men	7
Age, median (range)	62 (48–77)
Accommodation	
Living alone	8
Living with partner	10
Geographical representation	
Capital area (Stockholm)	12
South Sweden	3
Middle of Sweden	2
North of Sweden	1
Highest level of education	
Compulsory school	1
Senior High school	6
Folk college/university	11
Current occupational status^a^	
Retired	9
Working	9
Job applicant	2
Disability living allowance	1
Disability pension	1
Self-reported representation	
Physical limitation^b^	3
Mental limitation^c^	3
General population	4
Self-reported expertise area	
Physical limitation	1
Mental limitation	1
Health informants^d^	6
Previous screening experience	
Yes	16
No	2

^a^More than one option possible

^b^For example, vision or hearing impairment

^c^For example, autistic spectrum disorder, anxiety disorder

^d^From various countries

#### Findings from the Summative Content Analysis Approach

Participants generally had a general positive opinion of the DA. Many comments on paragraphs in the DA were as follows: “a clear message”, “well written”, “informative”, “simple and convenient”. At the same time, “no position taken” was frequently selected by participants on parts of the DA meaning that they did not have anything to say regarding the specific part of the DA. A smaller number of comments stated that the DA contained too much text. Comments on the language and difficult words or concepts were frequent. Examples of difficult words were “screening”, “decision aid” and “polyp”. It was suggested that the text should be reformulated, using a simpler language in general and especially for individuals with functional limitations, e.g. when speech synthesis is used. Suggestions for changes also included the importance of presenting information in different ways, using different languages and clickable links for those who are more interested. Other opinions included using social media and applying adaptions for those with visual impairment such as not using underlined text, but instead bold fonts or quotation marks. Furthermore, frames and gridlines can make reading easier for those with visual impairment, but caution is required regarding different text colours.

Worry or anxiety related to the content were present, in terms of being afraid on how to perform the test, that the information can lead to worrying thoughts, e.g. of having cancer and concerns regarding the colonoscopy examination such as pain and risks for complications (necessary to perform if the stool test contains blood). Thoughts like “do people read this kind of information” or “is this information necessary to be able to decide” were expressed and also “it is difficult to make independent decisions” and “difficult to know what one wants”. To make contact with the health care sector was discussed and the importance of patient organisations in this context. Health-informants expressed that among people with other background than Swedish, the permission to participate can be an issue in households where the tradition is that the husband decides if the wife can participate in screening. Preventive health care is not always known to people and can therefore be hard to understand.

#### Findings from the Conventional Content Analysis Approach

The analysis resulted in five categories (Table 2). The first category, Evoking emotions, concerned how the text, especially the words “cancer” and “screening”, may cause fear in some people, or that a faecal test could be embarrassing and not something one asks for help with. However, the text also contributed to a sense of relief, e.g. when reading that a polyp does not progress to cancer overnight. The second category, Text comprehension, included both positive and negative comments, such as long and complex sentences, e.g. too academic, as well as short and clear, while many participants appreciated the use of the word “dirt” instead of faeces. The third category, Text content, included comments on text passages that were not perceived as important for the decision, while some wanted more detailed information, pointing out that the text should be more attractive in order to motivate further reading. A majority of the participants stated that they had learned a lot by engaging in the DA. In particular, the health informants considered the DA a helpful tool when informing individuals with a foreign background about cancer and screening.

**Table 2:** Content analysis of interviews using the think-aloud approach

Category	Codes	Examples from interviews
Evoking emotions	Worry and anxiety	the word cancer is loaded too much information makes people worry many may think it is scary you can become a hypochondriac when reading this
	Relief	it calms me to know that cancer doesn’t progress overnight good to know that only a low % get cancer
	Embarrassment	it could be embarrassingly for women if men are around (when receiving the test) you might not ask for help with this kind of test
	Sensitivity	(the text) is a bit patronizing that could be sensitive for persons from other countries
Text comprehension	Too much text or to long sentences	no particular words just too long sentences unclear how many die from in cancer
	Too complex sentences or single words	difficult to understand the paragraph about prevention the language is a bit too academic
	Short and clear	brief information is good dots and short paragraphs are good stool is a better word then faeces as not everyone knows what that is
Text content	Text not relevant for the decision	the text about values is not important for my decision the text about screening is not important for my decision
	Lack of information	lack of information at the beginning of DA but it appears later on more details should be provided about what alcohol level means more information about cancer might be good
	New knowledge	I learned a lot by reading this DA as a health communicator this DA will help me a lot
	Make it attractive	the heading needs to attract me the text should increase people´s self-confidence to do the text
Motives for participation	Cancer in the family	more motivated when having a relative with cancer maybe more positive when having experience of cancer in the family
	Symptom of disease	maybe more motivated if you are sick
	Knowledge of cancer and screening	if you know that there is treatment you may become more motivated if I don’t understand that it is about me, I will not participate
	Someone to talk to	terribly important to be able to have a dialogue about this
	Faith	if someone professional tells me that this is good I trust it secure trusted sources are important (Schizofren) society needs to give guidance
Barriers to participation	Difficult to contact healthcare services	complicated to contact healthcare services nowadays when it’s digital as I am deaf, I can’t contact the healthcare services chat is only for the young (deaf) ones
	Culture	people get information from their home countries that may not be in line with this information in some cultures, men make decisions for all members of the family
	No one to talk to	as a deaf person you may not have many to talk to you may not dare to talk to your family
	Lack of support	difficult because my attendant (blind person) is only aloud if I am sick, not for prevention you may need help to take the test you may not have the confident to take the test, worry about doing it wrong
	Feeling healthy	tiresome to do the test if you’re not sick
	Suspicion	fear of being hung out on the internet fear of being traced (phone in flight mode)

The fourth category, Motives for participation, concerned experience of cancer in the family and having symptoms as a motivator for participating in screening, but also knowledge about screening and cancer was perceived as important. Someone to talk to about the decision and a trusted source or authority were also mentioned as important. The last category, Barriers to participation, was related to a feeling of being healthy and therefore in no need of cancer screening. The categories, Motives for participation and Barriers to participation, however, were related to disability, such as communication with healthcare services via digital methods, often impossible for people with visual impairment or mental limitations (see Table 1). No one to talk to was also perceived as a barrier to participation, especially for individuals with vision or hearing impairment living in small communities where people know each other, which leads to lack of integrity. Lack of support, e.g. the regulations state that attendants are only allowed for hospital visits when the person is sick and not for preventative purposes, which is problematic for persons with a vision impairment. Participants with mental limitations (please refer to Table 1) lacked support in terms of someone to help them with the faecal test or make them feel confident enough to take the test. Another barrier to participation in cancer screening in persons with mental limitations (please refer to Table 1) was the fear of being traced or “hung out” when using the internet, which is why they often turn off their phones, making them difficult to reach.

#### Findings from the Analysis of the Pictures

There was great variation in the range of opinions, emotional reactions and suggestions for improvements. Participants’ responses varied depending on whether the picture was abstract or more realistic by nature. Table 3 presents examples of the findings under the headings: Having a neutral outlook, Being in favour, Not being clear, Feeling scared and Suggestions for improvements.

**Table 3:**
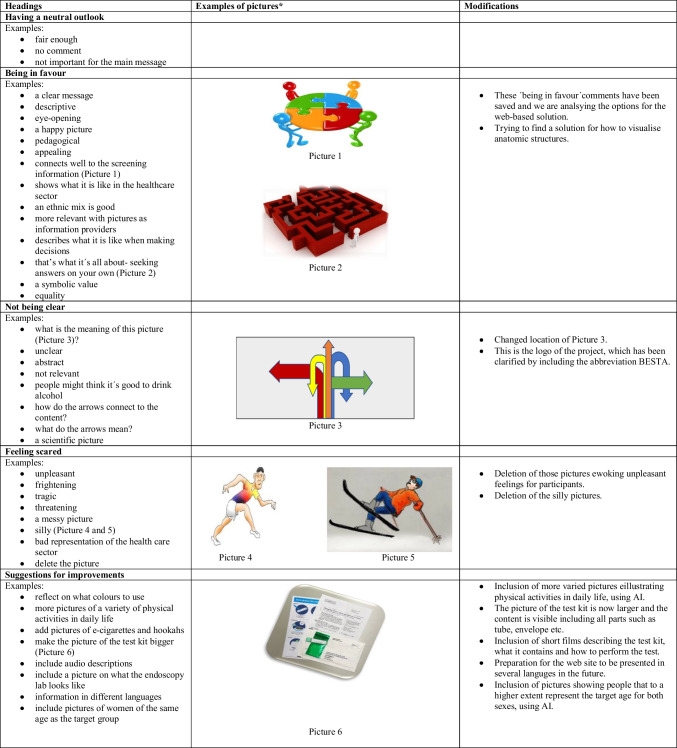
The analysis of the pictures in the decision aid and the subsequent modifications

^*^All pictures from this website: https://creativecommons.org/share-your-work/use-remix/. As they describe it on the website: “openly licensed creativity in “the commons” — the body of work freely available for legal use, sharing, repurposing, and remixing” (retrieved March 7, 2024)

#### Findings from the Analysis of Questionnaires

Some items were considered unnecessary, having an unclear purpose and/or were found to overlap. Participants also perceived difficulties with single words, formulations or whole sentences. However, they also suggested adding some items, for example “I have received information to be able to make a decision” or “Do I know what to do if my stool sample contains blood?” (Table 4).

**Table 4:** Comments on items from the questionnaires included in the decision aid and the subsequent modifications

Comments	Items	Modifications
Difficult words (advantage)	“I understand the advantages of cancer screening”	• Item changed to: “I understand why it is good to participate in cancer screening” • Inclusion of free text response option
Scientific language Purpose of question unclear	“I think support from the healthcare sector is important when making a decision about cancer screening”	• Item changed to: I want support from the healthcare sector (e.g. nurse, physician) to make decision on cancer screening participation • Inclusion of free text response option
Difficult wording	“It’s difficult to know if the advantages balance the disadvantages of cancer screening”	• Deletion of this item
Difficult to understand	“I think it is important that cancer screening doesn’t take too much time”	• Item changed to: “It’s important that cancer screening doesn’t take too much time”
Unnecessary question	”I’m not sure whether or not I will participate in cancer screening”	• Item changed to: “I’m not sure if I will participate in cancer screening” • Inclusion of follow-up item”What is it that you feel uncertain about?” with free text response option
Questions too similar	“I perceive it’s easy to use digital technology” and “I feel comfortable using digital technology”	• Item changed to: I find it easy to use the internet • Item changed to: I feel safe using the internet • Inclusion of free text response options for both items
More response options needed	“Do you smoke?”	• Inclusion of item: I think it’s important not to smoke” • Inclusion of “I don’t know” option • Inclusion of free text answering option
Free text response option needed	“It was difficult to answer the questions”	• Inclusion of free text answering option “Please elaborate”

#### Modifications After the Interviews

After finishing the analysis, the DA was scrutinised and modified, adapted and changed in accordance with comments from the participants. Sentences were made clearer and shortened, unnecessary words deleted. Certain words were changed, or an explanation added, such as for the word “decision aid” where support tool was used instead. For the word “screening”, the explanation-directed testing or examination was added. The suggestion to present information in different ways was applied, and short films were recorded about what the test kit contains, how to undertake the test and how to proceed while in the toilet handling the faeces.

For parts in the DA described as being a bit emotional (evoking feelings of embarrassment, worry, anxiety, lack of support), short interviews on making the decision, how to perform the test and the waiting period, from posting the envelope to receiving the answer, were conducted with a former participant from the general population. A chapter entitled *Frequently asked questions* was also added to the DA with questions relating to participants’ comments and concerns during the interviews. Examples of questions are: Can I phone someone if I have further questions? Do I have to be on leave from work? Can someone help to perform the test? Can I get any help if I don’t speak Swedish well? All questions are followed by brief answers, after which the user is linked back to the relevant chapter in the DA, or other websites such as The Swedish Cancer Society.

Comments on the pictures and questionnaires were taken into account and pictures deleted or modified; please see Table 3 for details on modifications. Regarding questionnaires, some items were added, others deleted or modified. Upon request from participants, a free text space was added for several items and more items added related to the chapter on lifestyle. Please see Table 4 for details of modifications.

After these modifications, all updated materials (the DA, FAQ, questionnaires, films and interviews, pictures) were sent to students at Nackademin (a Higher Vocational Education in Sweden), who, together with their supervisor, helped us to create the prototype of the website. We had continuous discussions, and modifications and revisions were performed during the process.

### The Alpha Phase

#### The Decision Aid (DA)

The prototype of the website was finalised during winter 2024. It includes seven clickable buttons with texts, short films and pictures and the content is structured under the following topics: To make decisions, Cancer screening, How to perform the sampling? Colorectal cancer, What happens if there is blood in the stool sample?, Values and attitudes and Lifestyle. The DA also includes self-reported questionnaires (on screening knowledge, values and preferences and lifestyle), an evaluation of the DA and frequently asked questions (FAQ). The DA is, in its current format, not publicly accessible.

#### Participants and Study Setting

Purposive sampling was used by multiple channels, including the snow ball method, but to a lesser extent [28]. Contacts made during the design phase was approached again in order to recruit new possible participants for the alpha phase. For instance, contact persons at various non-governmental organisations (NGOs) were e-mailed again; they reached out among their members and went back to us with names of interested individuals willing to participate in an interview. Furthermore, organisations working with social care were approached, such as The Salvation Army and Stockholm City Mission.

#### Data Collection

A pre-designed Excel® sheet was prepared and followed the structure of the website. The think-aloud approach worked as a basis for the interview, and they were conducted according to participants’ request online via Microsoft Teams® or in real life. The interviewer followed an interview guide but was at the same time flexible and adaptable to the participant. Interviewers were AJ or KF, and documentation during the interviews was done by JW or AJ. The prototype was shown to the interviewee chapter by chapter, and they were asked to think-out loud and provide their thoughts, comments and suggestions, which were documented accordingly. Sometimes, follow-up questions were posed, or clarifications were asked for. After interview number 5, it was decided upon not to go through all the questions/items in the (1) self-reported questionnaires, (2) the evaluation of the website and (3) in the chapter FAQ. This was decided upon since it took so long and was not considered relevant to scrutinise each question/item. Instead, the participant was asked on their perceptions and opinions in a more general matter, regarding the relevance of those parts of the DA. All participants were given two cinema tickets as a small incentive. Recruitment started during winter 2024 and ended in autumn 2024.

#### Data Analysis

The data analysis was performed in an iterative process, supported by aspects of *conventional content analysis*, as described by Hsieh and Shannon [34] and by a similar methodological approach applied in other research [35]. The material from the Excel sheet was divided between KF (the text paragraphs under each button, some films, some pictures) and JW (questionnaires, FAQ, some films, some pictures). The process started by organising the responses from participants according to content, i.e. a display of a text paragraph in the DA and underneath all responses from participants to that specific part. This was done in order to make the process more visually comprehensive and easier to follow for the IT consultants, helping out with the revisions of the website. After that, responses were condensed and coded and short summaries were written underneath each text paragraph. All the analysed material, including the summaries, was scrutinised and discussed among authors KF, JW and AJ, until consensus was reached. An example of the analysis process is displayed in Table 5.

**Table 5:**
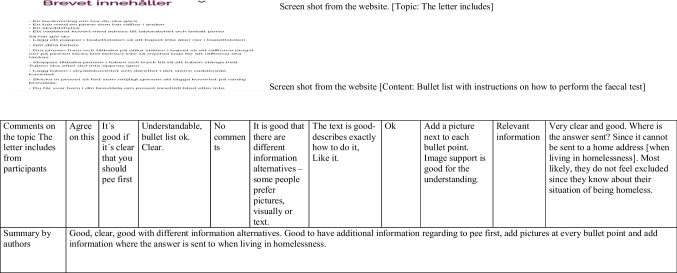
Example of the analysis process in the alpha phase

In order to address *digital accessibility*, the IT consultants were instructed to adhere to the Web Content Accessibility Guidelines (WCAG) [36], addressed by the Swedish Agency for Digital Government [37]. A separate analysis was performed on the specific comments from participants regarding digital accessibility, in relation to the text on the website. The comments were scrutinised and discussed among AJ and KF and revisions formulated.

The findings presented here are focusing on the texts under each topic (except for Lifestyle) and aspects of accessibility regarding the texts. The analyses of questionnaires, FAQ, pictures and films and the topic Lifestyle will be presented later on.

### Findings from the Alpha Phase

#### Background Characteristics

In total, 11 think-aloud interviews were conducted (April to September 2024). The participants chose the setting, and 9 interviews were performed online via Microsoft Teams® and two in a real-life setting. Interviews lasted from 70 min up to 135 min. Two participants had a sign language interpreter during the interview and one used speech synthesis. Table 6 shows participant characteristics.

**Table 6:** Background characteristics of participants in the alpha phase

	Participants *n* = 11
Gender	
Women	7
Men	4
Age, median (range)	64 (40–74)
Accommodation	
Living alone	10
Living together with someone (partner or children)	1
Geographical representation^a^	
Urban area (Stockholm)	10
Rural area	2
Highest level of education	
Compulsory school	1
Senior High school	1
Folk college/university	9
Current occupational status^a^	
Retired	6
Working	7
Self-reported representation	
Physical limitation^b^	3
General population	6
Self-reported expertise area	
Representative at the Swedish Association of the hearing impaired	1
Employed at the Swedish Association of the visually impaired	1
Employed at an autism organisation	1
Employed at an aid organisation	1
Previous screening experience	
Yes	10
No	1

^a^More than one option possible

^b^For example, vision or hearing limitation

#### Findings from the Text Analysis

The summaries for each part of the DA were sent to the IT consultants and they revised, updated and changed the website. This was an ongoing process between us (AJ, KF, JW) and the IT consultants. Examples of revisions included alternative wordings, such as avoiding using the wording “a letter sent to your home address” for those not having a home. Text paragraphs were shortened and/or presented in the form of bullet lists. Sentences were re-formulated in a more straightforward manner and clarifications sometimes added. Based on participants’ comments, the content hierarchy of the website was revised in order to achieve a more logical order and a website easy to follow and comprehend for a visitor. Please see Table 7 for examples of changes.

**Table 7:** Examples of revisions/changes made on the website after the alpha phase

Comments/suggestions from participants	Revisions/changes made
Unclear instructions when collecting the stool sample— add that you should pee first if you need to	One bullet point included in the instructions: “If you need to pee, do that first.”
Be consistent when using the words colonoscopy and/or bowel examination	Scrutinized the text so that the labelling is consistent
Unclearly described on how you will be notified that your faecal sample contains blood	Clarified and changed the structure on what will happen if the faecal sample contains blood
Unclearly described regarding risks with the colonoscopy	Added a new section and clarified the test regarding risks with the colonoscopy

#### Findings from the Analysis of Aspects of Accessibility

The revisions included by example the visual impression of the texts and how the website appeared for a person using a screen-reading programme. Revisions were sent to the IT consultants, and examples of those are presented in Table 8. WAVE®, a Web Accessibility Evaluation Tools, was used by the IT consultants. The tool can be helpful in making web content more accessible to individuals with disabilities. By example, accessibility and WCAG errors can be highlighted with WAVE.

**Table 8:** Example of revisions related to aspects of accessibility

Technique	
Coding	Revise so that a person who is using a screen reading program receives the correct and necessary information
Layout	
Colour	Keep white background colour
	Change to dark blue or black text colour
Margins	Apply left margins
	Be consistent
Drop down menus	Take away those menus from the website

## Overall Discussion

The specific aim of this study is to describe the *design and alpha phases* of the development of the individual digital decision aid for CRC screening and the perceptions of tentative end-users. Participants were interested in the project, especially individuals with self-reported functional limitations. They seemed happy to participate and contribute. Participants generally had a positive opinion of the DA and exhibited great interest in the research project. Similar findings were reported in a qualitative study on SDM in breast cancer screening including women of low socioeconomic (LSE) background. They (LSE women) perceived an online tool related to a SDM process to be of interest for facilitating meeting other women, providing access to healthcare professionals and information, when needed [38]. Nevertheless, opinions, emotional reactions and suggestions for improvements varied among participants, and we have tried our best to compile the findings and modify the DA accordingly. By example, with the short films, we hope to increase knowledge regarding the faecal test and decrease misunderstandings and technical problems with the test kit [16]. The evidence on the benefits of participating in CRC with a faecal test is clear due to the recent Swedish figures of 14% decrease in CRC mortality connected to screening [5]. The DA is still not finalised and the project continues in accordance with the IPDAS framework [22], Coulter et al. [24] and our previously published study protocol [27].

Regarding the text and formulations used, many participants preferred more readable text using a simpler language. Despite the attempts to avoid the use of medical terms, technical jargon and other discursive expressions in the text, participants reported difficulties. However, starting from the participants’ prerequisites might increase readability. This could be achieved by tailoring the information, simplifying medical terms and making the text appear similar, i.e. avoiding underlining, highlighting and italics [39]. Different perceptions regarding the texts and their appearances, including digital accessibility, on the website have hopefully been met after the alpha phase, and the iterative process will continue as we go along with further testing and development. For instance, certain words can be clickable and relevant links provided.

The comment “no position taken” was frequently used by participants in parts of the DA, for which there could be many reasons, including a perception of information overload or difficulties understanding the text and pictures. Another reason could be a personal fear of cancer and that the content of this DA evokes such feelings. However, the Cochrane review from 2024 on the topic [9] reports high-certainty evidence on the decision-making process and that a DA improves participants’ knowledge and risk perception, compared to usual care. At the same time, a DA decreases decisional conflict related to the perception of not being informed and the irresolution about personal values [9]. Similar to our intention, providing relevant information on the benefits and risks of screening should not be at conflict with screening participation [8] as stated in a study protocol with a similar aim to develop a DA for mammography screening in France.

Considering the choice and use of pictures, it seemed to be important for participants in this study to be able to identify with the individuals/characters depicted in the DA, for example concerning age and engagement in physical activities. Similar findings have been reported by others conducting research on decision support using pictures [40], emphasising the importance of considering recognition and representation when designing DAs. As our DA stands today, all individuals in the target audience are provided with the same limited set of images. However, with the increasing use of and possibilities offered by artificial intelligence (AI) in DAs [41], more tailored and personalised content, including pictures, may become feasible in the future. The students have partly utilised AI in their work with the website, e.g. to generate some, such as template pictures of people exercising or sketches of the bowel or a toilet seat. In addition, they have really made an effort to be inclusive and relevant for the target audience.

The work with the DA will continue with further analyses and later on beta testing; the DA is still under development. Nevertheless, in parallel with this work, we are designing a study that aims to evaluate the finalised version of the DA in a larger scale. This future study will consider aspects such as long-term usability, effectiveness, socio-economic factors, health literacy and decisional conflict, in relation to DA usage versus non-usage.

### Method Discussion

Using multiple channels, including snowballing [28], to reach individuals and especially those rarely included in research was successful and rewarding. Still, the recruitment periods lasted for quite a long time but since we aimed for a diverse sample, including individuals from groups deemed vulnerable, the recruitment became more challenging (lack of, or delayed responses, difficult to reach people etc.). The interviews resulted in rich data with detailed suggestions regarding future improvement of the DA and how it is perceived by end-users. The applied method, individual interviews including a think-aloud approach, appeared to be relevant for the present aim. Conducting the interviews via Microsoft Teams/Zoom was convenient and most often preferred by participants, even for those (*n* = 6) with special needs such as a sign language interpreter or their own visual aids or speech synthesis. Still, some participants preferred to have the interview face to face (*n* = 6), which we arranged. Data collection was terminated when sufficient and rich data containing both patterns and variations regarding the topic under study was considered achieved. Still, not all participants provided feedback on all parts of the DA, by example the pictures, where one reason being that it was difficult to grasp the pictures due to visual impairment. A few participants were outside the planned age range, either because we were not aware of it before the interview started or the participant was an expert whose perceptions and thoughts were valuable and thus included. We do not consider that including experts outside the planned age range diluted the focus since all inputs from participants were considered on an equal level. The *credibility* and *confirmability* [42] are considered strong as two of the researchers/authors (AJ or KF) performed all the interviews, and three of researchers/authors (AJ, KF or JW) were responsible for documenting them, meaning that they validated each other and furthermore applied *investigator triangulation* during coding, analysis and interpretation of findings [42]. In addition, all co-authors scrutinised the analysis and the formulated findings, before finalising the manuscript. However, as no audio recording was made, details could have been missed and/or misinterpreted. Nevertheless, we made the decision not to record the interviews for several reasons. One the one hand, we followed a detailed, pre-designed protocol aligned with the structure of the paper version/website. Any additional comments from participants were carefully documented to ensure that all critical aspects were captured. On the other hand, the choice not to record the interviews was influenced by the fact that our study involved considerable vulnerable individuals within the society. For instance, mental health conditions among participants could affect the sense of trust and security when deciding to take part in research.

The interviews varied regarding length, some participants presented many opinions and thoughts; thus, hardly any probing questions were needed, while other participants had almost no opinions or reflections, which is also important to address why we chose to include the summative content analysis approach [34]. The researchers performing the interviews (AJ, KF) have extended knowledge on the topic, and if necessary, clarifications or explanations could be given after the interviews had concluded. Participants were also encouraged to make contact if questions arose later. In addition, *reflexivity* [42] was continuously discussed between AJ, KF and JW during data collection and data analysis to shed light on potential preconceptions, biases and pre-understandings. In this regard, the pilot interview and the revisions made after it can be considered to add to the reflexivity. Finally, we consider the findings to be *transferable* [42] to other contexts or settings with similar preconditions and prerequisites as *thick description* was applied throughout the whole process, e.g. detailed field notes but also because of the nature of the decision, i.e. comparable to different screening programmes.

## Conclusions

Developing a digital DA is a complex process, for which a well-established framework, such as IPDAS and different expertise, stake holders and end-users are prerequisites. The variation in the findings and the variety in responses further illustrates the challenges involved in DA development. Nevertheless, the future work including further improvements of the DA and later on the beta testing will elucidate how the final version will be designed and perceived by end-users. The inclusion of individuals with both diverse backgrounds and functional limitations was successful and rewarding and should be an essential part of all research when relevant, especially if the goal is a more equal healthcare system, encompassing our culturally and linguistically diverse populations.

## Data Availability

Yes, available upon request.
